# Riggs’ Disease ; or, “What You Will”

**Published:** 1895-06

**Authors:** J. E. Cravens


### THE DENTAL REGISTER.

Vol. XLIX.] JUNE, 1895. [No. 6.

### COMMUNICATION.

### Riggs' Disease; or, "What You Will."

BY J. E. CRAVENS, D.D.S.

Read before the Mississippi Valley Dental Association.

"What's in a name? That which we call a rose
By any other name would smell as sweet;
So Romeo would, were he not Romeo call'd,
Retain that dear perfection which he owes
Without that title." —*Juliet.*

It would seem that a name should be first a designation, later, possibly, a definition, or in emergency a description. It is possible for a name to express too much. There was a time when a particular lesion, presenting certain diagnostic elements was generally known to dentists as *Riggs' disease.* Just what the proper name for that disease now is would be difficult, if not hazardous, to mention before this association. However, this one fact holds good against all mutations of discussion as to etiology, pathology and endless suggestions of endless names, in this, that, the disease under consideration continues to exhibit pus that exudes, or may be expressed from one or more aspects of an alveolus that has been enlarged by—what? Certainly not by pus; for who can assuredly declare that pus destroys bone? Lining the concave wall of bone that forms the greater limit of a "pocket" of Riggs' disease, or pyorrhœa alveolaris, etc., there is a sort of cushion of soft tissue, apparently highly vascular and protected by the socket periosteum, an arrangement that effectually prevents pus or any other of the contents of a "pocket" coming in direct contact with the alveolar bone. Truly, pus may bathe the involved portion of a root indefinitely, but does not destroy it. In the history of Riggs' disease (or if by any other name this rose of ours may bear a sweeter odor let it be so called), the only tissue really destroyed is alveolar process. But how? Realizing that a reasonable doubt as to adequate authority for some statements made in this paper may exist in the minds of some auditors, and claiming for myself a desire to induce others to careful study of all aspects of this subject, so important to dentists and their clients, I beg leave to offer some quotations from the Reference Hand-Book of the Medical Sciences, pp. 381 to 385, inclusive, *viz:* (Authorship of Dr. R. H. M. Dawbarn). "Inflammation of a bone may be induced by simple traumatism, by extension from a periostitis, by extension from arthritis, by exposure to cold, or to action of certain poisons, as phosporous, mercury, syphilis; by pressure, by eruptive fevers, typhoid, possibly acting as a primary, and certainly as a predisposing cause."

"It is of little clinical value to classify inflammations of bone, from an anatomical standpoint, into osteitis, osteomyelitis and periostitis, since primary periostitis, with exception of the traumatic and syphilitic varieties, is very rarely observed."

"Periostitis may originate from traumatism, either simple or compound, and in character may be simple (that is aseptic) or septic, from the presence of micro-organisms."

"Chronic, non-infective periostitis may be either fibrous or ossifying. In the former (fibrous) there is much increase in the amount of connective tissue, and the thickened membrane adheres unusually closely to the bone; in the latter (the ossifying) we have as a result an ossific deposit."

In the preceding quotations we have facts that appear to me to exactly fit the conditions manifested in pyorrhœa alveolaris; there prevails at first in the socket an inflammation of a "chronic, non-infective" character, that involves the periosteum and destroys the bone of the alveolus; later, under certain conditions, brought on by the surgical act, and supported by intelligent secondary treatment, the character of the inflammation of the periosteal membrane is caused to change from the "fibrous" and destructive to the "ossifying," which I hold to be an *acute* stage, and "we have as a result an ossific deposit." Dr. Dawbarn says further, "The new bone of inflammatory origin is not deposited in regular systems of lamellæ, probably owing to faulty nutrition, and it is sometimes absorbed and disappears."

I hold that the pathology here given applies directly to Riggs' disease; that by the same anatomical element, namely, the "thickened membrane," or what later is referred to by the same authority as a "granulation tissue," the spongy structure of the bony septæ is destroyed; and that after proper surgical procedure the "chronic" or "fibrous" is succeeded by the acute form, that accomplishes an "ossific deposit" differing from the true alveolar bone in that it is but a *bone eschar*. Thus it is that failure may occur from a constitutional tendency to revert to the "fibrous" form, and the new deposit may be re-absorbed, and pyorrhœa alveolaris reasserted. This last is failure.

Observation of cures of this disease, under my own hand and peculiar method of treatment, leads to the conclusion that if development of new bony structure proceeds until the "pockets" are filled, and so obliterated, and the new tissue shall be closely adapted about the roots (which it will be, if there at all), there will be no return of the disease, no recurrent absorbent action, even in cases that have been only partially filled in with this new "ossific deposit."

The author here quoted says further that the products of chronic periostitis are *pus*, *fibrine* and *serum*; these elements principally form the constituents of the fluid contents of pyorrhœa "pockets." Quoting still further, we have, "In periostitis and rarefying osteitis, the absorption of bone is thought by some pathologists to be caused by the presence of certain large, multinucleated cells, the myeloplaxes of Robin, called from this idea, osteoclasts."

Other pathologists disbelieve that these large cells possess any such power, and attribute the absorption to the influence of the new granulation tissues present in such cases, and lying in contact with the bone." I have already mentioned this new granulation mass or tissue and first referred to it as a vascular cushion or tissue that lined the greater diameter of a pyorrhœa "pocket" where it accomplished destruction of alveolar bone.

Dr. Dawbarn further says: "It is thought that the granulations (granulation mass) evolve an acid. Formerly it was believed that lactic acid was the solvent. Tilman's later researches seem to show that it is the carbonic acid contained in the blood which dissolves bone tissue."

In another place the author quoted says that sometimes no cause whatever can be assigned for periostitis; so it often is in Riggs' disease, that the cause or origin in a particular case can not be discovered or intelligently conjectured.

A significant point to all that I have quoted, and all I have read without quoting, on this subject, is that no mention is made of destruction of bone by pus.

The summing of my observations is, that *Riggs' disease per se is a periostitis of the "fibrous" character*, which Dr. Dawbarn has further described by the terms "chronic" and "non-infective" that this character of periostitis is destructive to bone and accounts to the dentist for the "pocket" that formerly bore so mysterious an aspect. Doubtless it is differentiated from that which occurs upon long bones or other parts of the skeleton, or under muscular tissue anywhere; because, this peculiar lesion occurs within the dental alveolus, within an articulation that is practically fixed although without ligaments.

Given an instrument sufficiently delicate and of proper shape for manipulating within these "pockets," and fingers in which sense of touch has been cultivated, and the velvety response of the cushion of "granulation tissue" that always lines these "pockets" may be readily detected.

In operating for cure of Riggs' disease, this "new granulation tissue" must be lacerated, for the purpose of inducing a radical *change from the chronic to the acute inflammation* of the socket periosteum, from which alone there is possibility and hope of securing the new "ossific deposit" that assures a cure. But in a "pocket" operation the complete removal of pyonal calculus and thickened and folded pericementum should be first accomplished, in order that the annoyance and serious inconvenience of certain hemorrhage may be avoided. The union of new bone with the root may be something analagous to anchylosis, or it may result in a true articulation similar to sympheses of the sections of the superior maxillæ. I am inclined to the latter opinion, because often the roots remain too loose for an anchylosis, long after every symptom of disease has disappeared.

A "pocket" and its contained pus, fibrine and serum, are simply results of the chronic periostitis of the alveolus, just as the calculus discovered on the root is a result deposited from pus; in all cases the periostitis of the socket must have an external origin. The most absurd of all the vagaries advanced as to the etiology of Riggs' disease, or pyorrhœa alveolaris, is that it has an internal origin, and begins at or near the apex of the root. It is always a pleasant thing to contemplate ourselves as specialists in medicine and surgery, with perhaps a dash of fine art and delicate mechanics, but our greatest danger lies in a possibility of becoming too highly medicated, too intensely scientific; this tendency leads away from a plain contemplation of the mechanical and physiological aspects of the disease under consideration. I have observed that a "pocket" may extend to and entirely around the apex of a root, after traversing its entire length, and far beyond the remotest deposit of pyonal calculus, and there be no external evidence of the existence of such a "pocket," nor entrance discoverable to it, save and except that peculiar bluish red color of gum characteristic, and the sense of an indescribable uneasiness felt by the patient; that the lesion may persist even after removal of all discoverable pyonal calculus from the root, and most careful antiseptization of the case; and that constitutional treatment fails to check the ravages of the destroyer.

So far as I have been able to discover, the pericementum has little or nothing to do with Riggs' disease, except, possibly, to be affected by it, and thus add an annoyance. It is doubtful that the pyonal calculus ever is really deposited upon pericementum, because a dead pericementum in the socket is impossible in a living subject, and the live membrane disappears as the calculus encroaches. The calculus being a result of the disease, may accumulate at a point rather remote from the cervix of the tooth, but there will be no pericementum left between the calculus and the entrance to the socket on that aspect of the root. The periostitis always is extended from an external origin or cause, such as an orbicular periostitis about the orifice of the alveolus, underlying a severe case of gingivitis, or an injury to the deeper membrane by dam-clamps, or badly adjusted apparatus for regulating teeth; ill-fitting crowns, independent or attached to bridge-cases; long protracted ligating at cervix; or, from traumatic injuries immediately about the socket; from any cause—as simple a thing as a splinter from a wooden pick thrust into the tissue of the gum—a frequent occurrence. Often the injury that sets up a periostitis is due to careless manipulation of dental instruments; and, as Dr. Dawbarn says of periostitis in general, the origin of Riggs' disease can not always be determined.

I believe that pyorrhœa alveolaris is not contagious; Dawbarn says that "chronic" periostitis is "non-infective;" this socket lesion is not a heredity, although conditions *may* be that tend to favor its establishment ultimately, and yet the disease may never obtain. It is not constitutional, and constitutional treatment does not facilitate its cure any more than it does the removal of an ingrown toe nail; it is not always symmetrical; it is not confined to adults, but as early as fifteen years has presented most stubborn resistance to treatment. While it may be associated with gout in Europe in many instances, in the United States it certainly is not the case, else the arthritis is most successfully concealed. This point has already been answered by Dr. A. W. Harlan, who declared that in this country gout is almost unknown. Riggs' disease often is associated with affections of the Schneiderian membrane, but prevails unaltered after that membrane has returned to a state of health. It certainly is true in the United States that a majority of victims of pyorrhœa alveolaris are also afflicted with nasal catarrh; but it could scarcely escape such association in a country where the catarrh is almost a badge of nationality.

Riggs' disease is most difficult to overcome in incipiency, that is, that condition just beyond gingivitis, where pockets are just beginning or hardly begun. In such cases the lesion consists in an orbicular periostitis, the operation for which was first described by Dr. A. W. Harlan, consisting in scraping the periosteum from the bone immediately around the orifice of the alveolus, and smoothing the bone, if rough; for which Dr. Harlan designed a pair of right and left chisels that still are the best adapted instruments for this purpose. I have found pyorrhœa alveolaris most easily cured in middle life, and always more satisfactorily treated where the "pockets" were fairly well formed. Very deep "pockets" are not readily dealt with.

Probably there was written during the last two years, on this general subject of pyorrhœa alveolaris, more than had been during a decade before. I was about the first to begin that season of activity in a campaign against this disease, which I did in a report of a case in practice before the American Dental Association, August, 1892, at Niagara Falls, N. Y., wherein was given all details of treatment of a case of nineteen pus "pockets," which I there declared to be cured, the treatment having all transpired during the month of July preceding. It was not to be expected that my statement would be accepted, and in that I was not disappointed; with the exception of Dr. Harlan, I believe every voice was against me there; the general sentiment was that the case was not cured, and that I was deceived by appearances; others were blunt enough to declare that it had not been a case of pyorrhœa alveolaris at all; one gentleman would accept no diagnosis that was not backed by analysis of urine of the patient. But I am used to that sort of argument and courtesy, and so survived to examine that case again in August, 1893, and found that there had been no return of the disease at any of the nineteen points treated the preceding year. I examined this case again in September, 1894, and still all was well with the places treated. Two years ought to be a fair test of treatment.

At the close of 1893, Prof. C. N. Peirce, of Philadelphia, proclaimed the gouty character of pyorrhœa alveolaris, ascribing it to presence of uric acid in the blood, and claiming in proof of this connection, that uric acid was to be found in the calculus of this disease. While not prepared to admit all that Prof. Peirce has written on this subject, and yet not prepared to deny, I trust not to do injustice to one whom I have long esteemed, and so will express no opinion on the uric acid and other propositions advanced.

The first paper by Dr. Peirce called forth an avalanche in response, which appeared in the *International Dental Practitioner*, early in 1894, and impelled the editor of the *Western Dental Journal* to remark, "Here we come, head us off, dad gast our fool souls!" or words to that effect. Not the least amusing aspect of this remark by the editor mentioned, was the fact that only a few years before he appeared in the American Dental Association proceedings arguing that pyorrhœa alveolaris is catarrhal. He has maintained a silence for some time, which I regard as ominous.

The discussion of the etiology of Riggs' disease might go on for another century, and all cases of the disease might be lost during the grand discussion from the loftiest planes. I hold that *one case cured* is better than a thousand essays and speeches upon the possible or probable causes of a disease that has been robbing poor humanity of good sound teeth, probably ever since the Ark rested on Mount Ararat. Of all the half score responses to Dr. Peirce's essay, only one suggested practical measures for the cure of the lesion.

In 1894, I had the temerity to write and have published a book, partly for the gratification of "mine enemy" but principally in a hope that many other brother practitioners would avail themselves of that system which had been so uniformly successful under my hand, in curing Riggs' disease. I still pursue this system with success, as a long list of grateful patients will attest. There are "none so blind as those who refuse to see." In Walter Scott's story of "Robert, Count of Paris," a character is brought to the light of day, from the cells under the Blachernal Palace where he had for several years been confined in total darkness; the poor prisoner for three days insisted that he could not see, and at last was induced to open his eyes and try; much to his surprise he could see. He had refused for three days to open his eyes.

President TAFT: The paper is now open for discussion. I will ask you to be prompt, gentleman.

DR. A. W. HARLAN, of Chicago, being called for by a number of those present, responded as follows:

Mr. Chairman, and gentleman: I am sorry I did not get in early enough to hear the beginning of Dr. Craven's paper; I thought it would not come off until tomorrow morning. Before I came here Dr. Cravens sent me a brief synopsis of the paper, which will probably furnish me with that portion which I did not hear. I would like to read the first paragraph of that synopsis, not for the reporter to take down, but simply to furnish a ground-work for my remarks.

(DR. HARLAN then read as follows: "Riggs' disease exhibits pus from one or more aspects of a socket or alveolus that has been enlarged by what? Not by pus, for who can say that pus destroys bone? Lining the concave bony wall that forms the greater limit of a 'pocket' of this disease, there is a sort of cushion of soft tissue, highly vascular, that is itself in turn lined and protected by the socket periosteum; so that neither pus or other of the contents of a 'pocket' can come in direct contact with the alveolar bone. Pus often bathes a portion of a root, but does not destroy it.

"In Riggs' disease or pyorrhea alveolaris, the only tissue really destroyed is alveolar process:—but not by pus. How then? In the essay itself I will give a number of quotations (necessarily incomplete) from a chapter by Dr. H. M. Dawbarn, In the Reference Hand Book of the Medical Sciences, Vol. V, pp 381 to 385, inclusive. All quotations therein are from this same author and chapter, and I use them to prove that Riggs' disease *per se* is a periostitis, of a fibrous, chronic, non-infectious character, destructive to bone and to the alveolar ridge most of all.")

The consideration of the etiology of the so-called Riggs' disease is a question that should concern every dental practitioner, because in nearly every locality in the United States we have loosening and loosened teeth. In that category we will not include teeth that are loosened by the mechanical arrangement of plates and clasps, or regulating appliances, but teeth that become loose from causes within the mouth, at least not mechanical. In the first part of the discussion on this paper I am unable to see how there can be a production of pus in any locality in the body, without the presence of micro-organisms. I am not aware that any sort of trauma, or any application of any corrosive agent to the natural tissues of the body, that are capable of decomposition, will produce pus, without the presence of micro-organisms. The author, I think, denies the production of pus from micro-organisms. I don't consider that Dawbarn is very good authority on that point. The experiments that were made some years ago by Herman Knapp and his collaborators show that the introduction of various irritating agents beneath the skin in sealed glass tubes and bulbs, when introduced under the greatest antiseptic precautions, and the bulbs afterwards crushed beneath the enclosed skin, that in not a single instance was pus produced, nor could the pus microbes be discovered in the serous exudates found in those cavities on opening them either at the beginning of the process or at a later period. The conclusions that were arrived at by Knapp and his collaborators at that time and to this day have not been disproven; and this I conclude, considering the large number of workers in bacteriology in the various departments of medicine and surgery, indicates that the scientific world has accepted this experiment as conclusive, up to the present time. The paper of Dr. Pierce and his school has been very violently assailed by Dr. George S. Allen and some other writers, with chemical analysis to show that uric acid was not found in the calculus that was taken from, or near, the apices of the roots of the teeth. So that, it seems to me, is still an open question. One chemist in Philadelphia found some urates, I think, and the chemist in New York did not find any. Consequently either the calculus was not the same, or one or the other of the gentlemen erred in making his analysis As a matter of fact, and in this respect I agree with Dr. Cravens, we find the so-called Riggs' disease at almost any age. He states in his paper that at fifteen years of age and upwards he has found it. I recorded one case about four years ago where I found the disease as early as the ninth year. I never saw but that one; but it was distinctly the so-called "Riggs' disease" pocket, and I called attention to it at that time. The trouble about the discussion of this whole question lies in this particular: we find teeth that are loosened and loosening, with deposits on the sides of the roots, and sometimes deposits covering the apices of the roots, say two roots of molars with the third untouched. We find deposits over the apex of a single tooth and a pocket, and the tooth not perceptibly loose; and in other cases we find something that is founded on so-called "Riggs' disease," where there are absolutely no deposits on the roots of the teeth—they are entirely free from deposits—and still the teeth are loosened, and when the finger is pressed under the gum, from the apex down to the gingival margin we find a discharge of pus. It seems to me that we should restrict the classification of this disease and make what we would call "Riggs' disease," a disease where there is always a deposit on the root of the teeth, or else confine it to where there is no deposit, but where the gum and periosteum is separated from the root. These two cases should be differentiated, because in the latter case it is hardly possible that the character of the pus that is produced through the agency of micro-organisms would completely denude the root of the tooth of a previous deposit of calculus. Is there any variety of pus so active in its nature that it will separate such a deposit from the root of a tooth? I, at least, have never observed such a character of pus. Taking then, that phase of the disease where there are deposits on the roots of the teeth, the question before us is, what produced this deposit? Is it of constitutional, or local origin? I will try and answer the last part of it first. If it is of constitutional origin why does it not deposit on the roots of all the teeth uniformly in the same mouth? If it is a truly constitutional disorder why do we find that there may be a first bi-cuspid with deposits, and then a second molar and a second bi-cuspid and the first molar entirely free? I do not know that that is absolutely proof positive that it is not of constitutional origin, yet you see hundreds of cases where a so-called "Riggs' disease" has attacked a tooth, and you find two teeth, or three, or four on one side of the mouth, or all the teeth in position, not loosened or loosening, and the teeth on the other side perfectly free. Have you not seen such cases? After reading the paper of Dr. ——— of Esen, Germany, in 1881, on what he called infectious alveolaritis, I have been strongly of the opinion that the so-called "Riggs' disease" was a disease of local origin, but that constitutional complications might exist which would enable or permit this disease to progress more rapidly and be coincident with it. It is not impossible; but with the inception of the disease, as a disease it seems to me that there is no evidence to support the theory in spite of all the papers of Miller, Black, Wetzel and others—in spite of all the papers pro and con, that there is no evidence to support or prove that it is a disease of strictly constitutional origin; but that it must have some external agencies to begin with, no matter whether those be from the effects of a clamp, or a pair of forceps misapplied, or an unskillful operation around the tooth, that it must be local.

In the next place, Dr. Cravens, as I understand it—if I am wrong I wish to be corrected—says that it is not infectious. Now, a gentleman who stands very high in the bacteriological world, whose name you would recognize as deserving of that high position that he holds, if I mentioned it, communicated to me in confidence a case of infection that he brought about purposely to determine the effect in the mouth. He said that he did not wish to have this published at the time, because he was not sure of it; but, later, after the tooth was lost through the infection, he told me that he was satisfied that it was infectious; and he still claims that it is infectious. He claimed that the introduction of an instrument bathed in the pus from a "pocket" of this kind, if used on a sound gingival margin or beneath it, would carry the disease in the same mouth from this pocket to another, as he had seen a case where the teeth had been thus lost.

If I am not mistaken Wetzel also inoculated one or two or more dogs—I think I am not mistaken in that—to prove the infectiousness of it. Now, if we accept the theory that pus cannot be produced without the agency of micro-organisms, we must accept the theory that the so-called "Riggs' Disease" is infectious; otherwise the first part of the theory would be useless.

The next question would be, if a nidus were formed around the root of the tooth for the collection of the spores of the pus microbe, naturally the destruction of the peri-dental membrane would be first, and secondarily the destruction of the alveolar socket would ensue, and, thirdly the production of the deposit would result; that is, the three processes are, first the peri-dental membrane destroyed, and the pericementum; second the bony socket contiguous; third the deposit from the serum, sanguinary pyonal, as Dr. Cravens calls it. Now, the mere deposition of granules of this sort of calculus being merely mechanical is not sufficient in itself to produce pus, because we deny that pus is produced without the agency of micro-organisms. Second, the chemical constituents of deposits found on the roots of teeth under such circumstances are found to be different from the ordinary salivary calculus, because it is wanting in certain constituents that are found in the ordinary salivary calculus as we find it. Another proof of the fact that it is not of salivary origin is this: that beyond the line of detachment of deposition of salivary calculus you will never find a peridental membrane recede from the root, and as soon as you dislodge the salivary calculus from the root of a tooth down to the extreme point of its deposition you won't find any pus corpuscles, or pus microbes; because if you did, there would be a detachment beyond, that would be a nest for the future proliferation of developed microbes, which would destroy the peridental membrane. We don't find the peridental membrane destroyed beyond this mechanical deposition of salivary calculus. It is deposited on the roots frequently after the peridental membrane has been destroyed by the so-called "Riggs' Disease," and we find salivary calculus on the roots of such teeth; but in a case of that kind it is just as though you could run a white-wash brush over the side of the root, and it leaves it ragged and irregular, in granules, there being no membrane around the root to jut up against it; the membrane does not extend to and around the apex of the root only in exceptional instances. The destruction of the bony socket of the teeth may be accomplished through the ichorous nature of the pus, produced by a certain micro-organism called the micrococci pyogenes citreus. It is possible to produce the death of the bone surrounding the root of a tooth, and a number of observers have found that it was necrotic tissue, and that it was an organism that could be isolated from the pus. There are some teeth, where the apices of the roots have been uncovered through the proliferation of the so-called "Riggs' Disease" and we find that the pulp dies in consequence, and that the root of the tooth assumes a bluish-black hue, and that the cementum on the roots of such teeth appears to be totally devoid of any semblance of vitality. This is only in extreme cases; and the question in my mind would be in differentiating, whether that cementum was destroyed in consequence of the presence of the pus, or whether it acquired that character from the destruction of the pulp. That I have not been able to determine. I should be inclined to the belief, that the vitality of the cementum was lost simply on account of the destruction of the pulp, after the peridental membrane was stripped from it. Dr. Cravens says that pyonal calculus is a result of the disease; I am perfectly willing to agree with that, because as I view it, as I have stated it, it must be the result of the disease; and I differentiate it from salivary calculus, as I have previously stated; and I say that it is the third step in the natural history of the disease, if left alone.

I don't know that I am called upon to say anything about the treatment of this, as I believe it is simply the etiology we are now considering, and we will leave the treatment alone. I will only say this, that if it were a constitutional disease we could cure it by simply removing the mechanical difficulties in the way and then administering our remedies constitutionally and the case would get well. But they don't get well without local applications. If the man is suffering from some grave disorder that requires the administration of remedies, why, that could be carried on at the same time; but the administration of the remedies, except for the toning up of the general system will not affect the cure in these cases, without the application of local remedial agents, whatever they may be. I think that is all I have to say.

Dr. J. S. Cassidy, of Covington: I do not think I have such pronounced views upon the nature of "Riggs' Disease" as to be unable to agree with many of the points made by those who argue pro and con, especially some of those made by Dr. Cravens, in his excellent paper. As Dr. Harlan says, there are cases and cases of this disease, and that all which are diagnosed as "Riggs' disease" are not truly genuine "Riggs' disease," or pyorrhœa alveolaris. Ever since Dr. Ingersoll suggested sanguinary calculus, near the apex of the root as the possible incipient stage of this disease, I have had an opinion that such was the case—that the disease was a local reflection of at least a constitutional predisposition to it. Now, while at this point I would say that Dr. Harlan asks why, if this disease be constitutional, all the teeth are not similarly involved? It is because, I think, if it be constitutional, that the conditions—the local conditions—in each tooth are not alike; that there is a law of periodicity, which applies to this as to most other things, all other phases of matter, animate or inanimate—a periodic law. That might be, for instance, I will not say proven, but as tending to the idea that there is such a law—instanced in this: why will a bicuspid withstand the destructive processes when its neighbors on either side of it have been destroyed? Why has it stood sound, alone, for so many years? And its mate on the other side of the mouth, say, first or second bicuspid, or any other tooth, if it has a mate, which it generally has, I believe. Those two teeth remain sound and free from disease until a certain period of life, nine years—which is the extreme limit of this disease (Riggs' disease), I believe, to appear, in seventy-five years, as the case may be; then disease appears, caries of the surface of the bicuspid, we will suppose, and it appears almost simultaneously in an analogous position on its mate. Now, why are those teeth persistently well so long, while all their neighbors have gone? Simply because, according to this view there is a periodic law to regulate these matters, as well as local influences. I have regarded this disease, called pyorrhœa alveolaris as a constitutional or as a local reflection of a constitutional cause; and I say that ever since the statement of Dr. Ingersoll, about ten years ago, of sanguinary deposition on the roots of the teeth, that I have regarded this disease as due, primarily, to these deposits. They are too minute for us to discover, even if we extract the tooth in the beginning—too minute to discover but yet of a sufficiently irritating nature to produce inflammation and its train of symptoms and the troubles that follow, and which have defied the best efforts of therapeutics to overcome. Now, what is the solution of that? Is the alveolus involved? I don't know. It might be, as Dr. Harlan suggested a deteriorated pus—not pus by itself, but deteriorated. I cannot conceive of pus alone causing the deposit, and agree with the essayist in that respect. I cannot agree with the essayist, however, that the calculus is pyonal, that it is caused by pus, cannot call it by that name. He gives it that name. Why? There is no reason why it should have that name. I do not think it can be called, properly speaking, pyonal pus. Pus is a consequence of the destruction of the disease itself. Micro-organisms, of course, must be present as a consequence of the disease, a catabolism instead of a metabolism; and why nature should attack the aveolus instead of the root of the tooth, is because, I presume, it finds the alveolus more susceptible of attack, more easily destroyed than the root itself; but if the root be extracted the disease is cured. My opinion is that a genuine case of "Riggs' disease" cannot be cured in any other way. I believe I have said all I wish to.

DR. HUNTER: The idea of my being inflicted upon this Association for the discussion of this subject is somewhat in the nature of a joke. However, there are several reasons why I have been selected by the committee. First; because I am personally acquainted with the essayist, and have a respect for him; second, because I am personally acquainted with the committee and have no respect for them; and, third, and I suppose the most important, is, that I know nothing whatever upon the subject! But in this day of advanced science, when the theories of yesterday are scorned today, and when the theories of today will be laughed at tomorrow, it matter little what is said on any given subject from either side for everything goes! When beef is considered a stimulant, and whiskey a food, the theories of yesterday are certainly not "in it" today. Our friend the essayist has given us a very plausible theory. I have had the pleasure, recently, of reading his little book that he refers to, in which there is a beautiful theory advanced, and in some respects a plausible theory. One of the plausible points of the theory is just, in my mind, what Dr. Cassidy objects to—pyonal calculus. He demonstrates in that book by analysis, and giving the authorities, that pus contains a greater proportion of lime salts than saliva; and he says in the book the wonder is, not that there is so much deposit from the pus as that there should be so little. To my mind that seems plausible. The constituents of pus, as far as the lime salts are concerned are very much greater than that of saliva; but Dr. Cravens is very enthusiastic; he gives us theories; he has given us a theory before. There are many of us here that remember how enthusiastically he advocated the theory some years ago of nature appropriating from the topical application of oxy-phosphate of lime material for making phosphate of bone. I believe he has given up that theory. I hope that this present theory will stand the test of time better than that did. In his treatment by mechanical manipulation he is certainly more thorough than the majority of us are; and we do know that in the treatment of this disease that the more thorough the treatment the more good we do the patient; but in my own mind I doubt very much if the removal of the result of the disease is going to effect a cure. You must get at the cause of that disease. We are endeavoring to do it, and the theories advanced by the essayists and others are all tending in the direction of discovering the cause of this disease. We need such enthusiastic observers in the investigation of any subject. The Doctor's hair is a little thin on top; but it is of a good color! I hope that his theories will stand the test of time. He claims to have produced cures by simple mechanical manipulation that the rest of us have been unable to do. In that book he speaks of a thorough removal of this calculus; and anything short of that is only a temporary success; or as he expresses it, a partial failure, I believe—(Dr. Cravens: Modified failure). Well, I am willing to admit that I have only made modified failures in all of my experiences. I cannot cure this disease; and I believe that the evidence is sufficient, from what the essayist says that it is hereditary, or that heredity is the predisposing cause.

I have seen too much of that in my own family—in myself. I have the disease in my own mouth, and I know a little about it practically; but cannot cure it. I guess I will put myself under the care of Dr. Cravens.

Dr. O. N. Heise: I did not have the pleasure of hearing the paper, but from the synopsis sent me I cannot help but agree with the main points, except that it is periostitis, and he does not say how that is produced; he simply makes that statement without saying what grounds he has for it; I wish he would make some explanation of that.

Dr. Cravens: Did you hear the paper?

Dr. Heise: I did not. I simply judged from the synopsis you sent me. Not having heard the paper read I don't think I am able to say anything about it.

Dr. Taft: Are there others who wish to take up the question, or make any general remarks on this subject?

(Dr. Wright was called for by several members.)

Dr. Hunter: He don't know any more about it than I do.

Dr. Wright: That is the reason I want to speak on it. I don't know that I can say anything of interest on the subject at all. The one point of the infectiousness of "Riggs' disease" I want to speak of as a matter of history. Thirty or thirty-five years ago, in Dr. Keely's office when he had the first case, we did not know anything about it, that is, the simple loosening of the teeth that was present, and existed then, as now. We believed then in extracting those teeth, so that possibly the other teeth would not be lost, believing that it was infectious from one tooth to the other in the same mouth. The disease used to spread; and we extracted the affected teeth for the purpose of curing the disease. That was thirty-five years ago and we don't do that now.

President Taft: Has Dr. Cravens anything further to say on the subject?

Dr. Cravens: I have very little further to say on the subject. Dr. Harlan stated that I said the calculus was the result of the disease. I see a rather quizzical expression in Dr. Taft's eyes, as though he wondered why I should find it necessary to make such a statement. I will explain that I should not have thought it necessary to lay down that for the information of Dr. Harlan, in the paper, but I made that statement because I know there are many, perhaps the majority of dentists, who have the idea that calculus is the cause of the disease, so that I took the trouble to explain that the calculus is the result of the disease, and not the disease the result of the calculus. In regard to the infectious character of the disease, if it were infectious it seems to me that it would prevail generally in any mouth in which it had once obtained a foothold. It has only been a matter of ten days, or two weeks, since I had the opportunity to examine the lower molar of a gentleman that I had treated two years ago. It was the only case of pyorrhœa in his mouth. I can not find a symptom of it now. He has pretty near a full complement of teeth. I met him on the street a few days ago, and as I was coming up here I told him I would like to look at that tooth; and he came to the office, and there was no sign of it anywhere else in his mouth. Now, Dr. Cassidy spoke of the calculus, and did not believe that I had any authority, or perhaps thought I was wrong, in holding that it was deposited from pus. I did not say it was caused by pus, but that it was deposited by the pus. Dr. Hunter came very nearly to answering that question; in fact, I think he did, but in his analysis did not give quite all of it, in his analysis from the book. It is shown by the authorities I quoted from, that the pus contains 300 per cent. more of the elements of the calculus than the saliva. So that is why I used the remark that I did, that Dr. Hunter referred to—I mean as to the salts. The other constituents that come from the pus we do not expect to find in the saliva. As to the extent to which the disease is curable, of course Dr. Cassidy's remedy is older than Dr. Cassidy—perhaps older than the disease.

I carried the disease back to Mt. Ararat, and would not like to go back further, because I believe some of our scientists claim our diseases have altered nowadays, because the solar rays did not come direct to the earth before the flood, on account of the constant canopy of clouds, the atmospheric conditions, too, were different, and the diseases before the flood were very much different. So I would not like to go back any further than the time when the ark rested on Mt. Ararat for the first case. In regard to inoculation, Dr. Harlan thinks some cases have been reported of that, and that there were evidences to prove that the disease could be transmitted by inocculation. I don't know anything about that. You may have pyorrhœa on one side, a lower incisor say, and it will not affect the other side. I have treated such a case and cured it; there was no more pus, no more congestion, and it remained in that condition and the others remained unaffected. It is not symmetrical, and that is one thing that proves to me that it is not constitutional. If it was constitutional, why would it not be general? In very many cases it is isolated, one tooth here and there—not symmetrical.

On motion the discussion on this topic was now closed.

PRESIDENT TAFT: We will now hear a paper by Dr. J. R. Callahan, of Cincinnati, entitled "English Tube Crowns for Bridge-work."

### ENGLISH TUBE CROWNS FOR BRIDGE-WORK.

We will admit, that modern bridge-work when properly constructed, is a blessing to suffering humanity; but great as the blessing may be, we have to acknowledge that there are many weak points that even the expert is unable to overcome

The man that will show us how to make the attachments to roots, that will not irritate the gums, that will not afford lodgment for food, that will not make a disgusting display of gold, that will not break or twist out of shape, that can be repaired without destr ying the whole piece, and can be adjusted without destroying sound teeth; that man will confer a great blessing upon our profession as well as upon thousands of people who carry about in their mouths those unsightly, broken and stinking abortions that are altogether too common in our midst.

It is our desire to call your attention to at least a partial correction of some of the defects in bridge-work as constructed in this country. The points in favor of the use of the English tube crowns in the construction of bridge-work, that we wish to demonstrate to-day, are the concealment of metal, the introduction of crowns that are easiest to mount, least liable to fracture, that do not change their color during the course of construction of the bridge, and that are easiest to replace in case of accident.

We will give a few details for the construction of a simple piece of bridge-work extending from first superior molar to first superior bicuspid. The abutment teeth are ground to the proper shapes; gold caps are then made to fit these stumps; after caps are in place upon the roots, cut a hole through the cap just over the root-canal for the pin to pass through, being careful to have opening of such dimensions that the pin wire made for these teeth will fit in the cap snugly, having selected a suitable crown fit it roughly to the cap; place the pin in the root and try on the crown; if it be much out of line with the other teeth the fault must be put right by bending the pin or reaming the canal in the direction necessary; try on the tooth once more; when the proper position for the pin has been ascertained, remove cap and pin from the mouth and solder the pin in its place.

(Being careful to use smallest amount of solder and to get perfect attachments.) The caps should now be put in position and the crowns placed loosely on the pins; the crown to be suspended between molar and first bicuspid is now fitted to its place; the neck or that portion of crown coming in contact with gum, should be ground to the right shape; a very thin platinum plate should now be soldered to a piece of pin wire of proper length; pass the wire pin through the tube in the crown and burnish the platinum plate to the V-shaped portion of crown just referred to; now try the crowns and caps in the mouth, place a little sticky wax on the second bicuspid crown and platinum plate and force all the pieces to place in the mouth; with a camel's hair brush place a very thin film of oil over the porcelain crowns, then remove the whole bridge from the mouth by means of plaster in the impression tray, trim the plaster from about the metal parts, then cover the pins and caps with impression compound, allow the compound time to become thoroughly cool, then cut away the plaster and remove the crowns; the oil that was placed on the crowns will make this easy; this will leave the pins sticking from the modeling compound; now place a mixture of plaster and marble dust over these pins and after it is dry remove the modeling compound by means of dry heat; this will put the metal parts in position to be soldered together and the porcelain crowns are out of the way, or in other words the piece will be soldered together without subjecting the porcelains to the heat that so often cracks and otherwise injures them; place a piece of platino-iridium wire extending from molar to first bicuspid; in the depressed platinum that was on the second bicuspid crown, flow enough solder over this to make a strong and smooth union; cool and remove and finish; place the metal on the roots in the mouth, adjust the crowns on the pins and see that everything fits to its place; then finish grinding the occluding surfaces; then remove the whole piece from the mouth, roughen the pins slightly with a fine file, then boil all the parts in acid to remove every trace of oil or grease, dry with alcohol; then we are ready to attach the crowns to the bridge; have a small vessel filled with melted sulphur, with pliers grasp the bridge by the metal pins, the crowns being in position, warm the bridge piece slightly in the flame of bunsen burner, then with a warm wire carry the melted sulphur to the pin in the grinding surface of crown; the sulphur will run into every crack and crevice in the neighborhood, and when cool the bridge will be ready to be cemented to the roots, and we will have a bridge piece that will show but a small line of gold at the gum border; that has been easy to construct.

The tube crowns are stronger than other kinds of porcelain teeth, for the reason that they are supported over their whole lower surface and the greatest strain in occlusion falls mostly in a vertical direction upon the crown and parallel to the line of the central pin; whereas in a flat tooth, the attachment being on one side only—the impact of the bite is more unevenly distributed, and not least of all they have not been exposed to the heat of soldering, which so often cracks and weakens the teeth in all kinds of metal plate work; the crowns under this method can not change in color. (1st), on account of their having been kept from the heat, and (2d), because there is no metal backing to contend with; and if a crown should be broken, a new one can be put on in a few moments, without removing the bridge from the mouth; I have yet to see the first one break. These crowns can be gotten either through your regular dealer or from C. Ash & Sons, No. 30 East Fourteenth St., New York City.

To all who are not familiar with this kind of work, and desire to give it a trial, I would advise them to read the paper of Dr. John Girdwood, in the printed proceedings of the World's Columbian Dental Congress.

The Dental literature of this country, will always be painfully silent on the use of these crowns, so long as the combination of Dental Manufacturers hold the American Dentist by the throat, as it does to-day.

Dr. Callahan having concluded his paper, President Taft called upon Dr. Ames, of Chicago, to open the discussion upon the same.

Dr Ames: I suppose that Dr. Callahan asked me to discuss this subject because at different times we have together discussed to a considerable extent this particular class of work and to the desirability of concealing gold to as great an extent as possible. I think that one of the most desirable objects to be attained in the construction of crown and bridgework is more scientifically artistic results than those we so often see. And it is to be done with the maximum amount of porcelain and the minimum amount of metal. I don't know of any better way to accomplish this than by the use of these English tube teeth, and in the manner described by Dr. Callahan; unless it be by means of the use of another form of teeth made by the same people; that is, the diatoric teeth which have an opening partly through the crown, instead of all the way. In my use of these teeth, as well as of the diatoric teeth, I sometimes have to proceed rather differently than as Dr. Callahan describes here, in order to get my crown, and the proper alignment of crown on the root. The Doctor says he fits the pin to the root, and if, by placing the crown upon the pin, it does not come in a proper line he reams the root and bends the pin. I am not always able to accomplish it that way. In a majority of cases I make the pin in two parts. To be explicit I would fit the band and cap to the root, perforate the cap, put the pin, which is to be contained within the root and solder it, and cut it off flush with the surface; then I would take an impression of that, as in an ordinary case of crown work—or without an impression work to the mouth direct—and grind my tooth to the position, giving it proper alignment, and then burnish and fit to the crown end of the porcelain tooth a cap, turning it slightly over the edges and perforating that at the point of the whole in the tooth, putting the pin in there and soldering it, and then put the two in their proper relative position, and run solder between the two, in that way getting a proper alignment where I could not do it nicely otherwise. It seems to me by using the separate piece of metal, one cap for the root and one for the tooth and burnishing the metal over the edge of the porcelain slightly, having the porcelain tooth in a sort of a bed, which gives it a more substantial attachment, I think, than if it is simply set together on a plane surface—it is in a sort of a receptacle, you may say—and I think it will stand a greater amount of strain. The diatoric teeth, I like a little better for bicuspids and molars. Dr. Callahan suggests that I draw a diatoric tooth on the black-board. I think you are all familiar with it. I am not a draughtsman. (A voice: You are a drawer.) Sometimes, if I am lucky!

(Dr. Ames then drew upon the black-board an illustration, and made remarks accompanying same which it was impossible for the stenographer to follow and re-present here in an intelligible manner. He concluded by saying:) I think the first work I did of this kind was with the English Tube teeth, because I happened to have a few of those before Mr. Sykes established the branch of Ash & Sons in this country; but when he came here I became familiar with these diatoric teeth, and I began their use, because, I think, you can get better patterns, better selections of these, while they do not carry much of a selection of the tube teeth. It must be five or six years or more; and I find that while at first glance it often seems that the attachment of the porcelain to this cap would be weak and not be satisfactory, yet from actual experience I find it is very strong. (A voice: The porcelain will break: can you replace it easily?) I never had one broken. (A voice: What would you do if you did?) I think you will find that they will hold; you can grind this hole closely, and if it gets too large simply fill in. I have never had one come off. I have never had the porcelain to break or come off from its attachments; but I have had the attachment to the root weaken. I have in my pocket a tooth that is made up in this way. Here is a molar of that kind, which was a dummy in a bridge piece that I changed, for reasons not necessary to mention, and it will show what can be done with those, and show about what I would consider to benecessary as to strength of gold. This was, of course, the dummy between two crowns, and will show about the way this can be done. That is attached upon the gold with silver. I find that these teeth, either the tube teeth, or the diatoric, are extremely satisfactory for these dummies, for making the span you can arrange these any place. Say you have, furthermore, two or three bi-cuspids, you can arrange them in position and flush up the backing here to a sufficient extent to give strength for the span. (The Doctor then explained certain peculiarities in the sample exhibited, and continued:) The advantage in the use of this English porcelain that makes it very desirable to me, is, the peculiar texture and homogeneity of the texture and material, enabling you to grind it down and polish it; and the advantage in doing your work in this way is, that this peculiar porcelain is liable to change color in firing, and in doing the work in this way there is absolutely no danger of getting the color affected. While speaking of this, I want to mention a matter possibly a little out of the line of this discussion: I want to speak of the possibility of using these same teeth for cuspids and incisors, in such a way as to avoid having the porcelain in position while doing the soldering, thus avoiding the danger of change of color. This is a method that has become popular among men who endeavor to do this kind of work nicely and in an artistic manner; in backing up incisors or cuspids, and then removing the porcelain from the backings; remove the porcelain teeth from the backings and insert through the holes in the backings little graphite pencil points, waxing these in position as the pins would be if the porcelain tooth were there, flowing the solder off the finger around these points, drilling out the cavity, the whole being retained by these graphite pencil points, cementing or fusing on the silver and rivetting down the pins. I have used that method; and I want to give the credit for my knowledge of that to Dr. Buckner, of Baltimore, Md., who gave me my knowledge of it, I think, in 1889. I have practiced it very satisfactorily since, and inasmuch as that has been written of and shown to a considerable extent recently, the credit of it should be properly placed; and so far as I know Dr. Buckner was the originator of that, as I obtained the idea of him at least six years ago. I think that is all.

Dr. Custer: I don't know that I can add very much to what has been said. I can only agree with the author of the paper, because he has given the idea and principles of bridge-work, when correctly constructed, having reference to the requirements of strength and hygiene and beauty, not omitting the element of repairability. The point of strength, I think, has not been touched upon in this: where a piece is suspended between say, the first bi-cuspid further back to the second molar, you would have, then a bi-cuspid and molar. There is the principle of suspension. As any of you know metal will stand more strain in a pulling direction than in a bending. We gain more strength at the least expense of metal by this treatment, the tooth being set upon a pin, and the pressure being brought to bear so that the effect upon the gold is simply a pulling strain from one abutment to the other. Another point of strength can be mentioned, and that is, let the teeth be put there on the principle of locking; have a groove ground in the side of one porcelain tooth, and the one that fits into that have a corresponding projection to occupy that groove in its neighbor; let that principle be carried clear along through the bridge; then if the pins were to be broken in any one tooth, I apprehend that the tooth would be retained by this locking of one tooth into the other. A point in regard to hygiene is, that the whole space is filled up—the inter-dental space. That is a necessary thing to observe where two teeth are in touch with one another, and the proper thing, of course, always to observe in filling. Here we have, let us say, three or four teeth, and the inter-dental space partially filled. Why not fill the whole space; let it be filled solidly. Have either platinum or pure gold next to the mucous membrane, and then block in solidly with pure gold teeth, at the outer surface imitating the surface of those teeth. The mouth has always seemed to be a self-cleansing thing; yet it is anything but that in many cases coming under my observation, If we are going to fill in there, why not fill it all in with a material which will not deteriorate, and which will not invite micro-organisms? And then I think we have done the best thing possible. Another thing, the base being of pure gold or platinum would not come in contact with the mucous membrane produce irritation. During mastication, especially, the food would not be forced in; normal action will keep that self-cleaning. If you have not accurately fitted that case, so that it comes in contact with the mucous membrane, the mucous membrane will fill that in after a time, next to the band. I think that is the most beautiful piece of work that can be constructed. There need not be but a mere line of gold on the outside. The point has been touched upon that these teeth may be ground, without marring their appearance, afterwards being polished. The point of repairability is also a strong argument in their favor. It is such a strong one that it is of the highest value, and it is the first recommendation that directed my attention to the use of these teeth; but I have never had to repair them. If necessary it is evident it can be very easily done, by the mounting of new teeth upon the old pin.

Dr. Emminger, of Columbus: I know so little about these English tube teeth it is hardly worth while for me to attempt to say anything. Some time ago Dr. Callahan wrote me and wanted me to discuss his paper, and said he would forward me a copy of it. That is the last I have heard of it. I came to the conclusion that he was simply making another effort to maintain his reputation, that he has so long enjoyed in this community, among dentists; but the manner in which he has presented this case, I think, displays some very good features. The possibility of constructing a piece of work which would have very little metal exposed, and also be easily repaired, when it becomes necessary, seems likely; but I have found in a number of cases of bridgework that it was not this part that needed repairing; you need new piers for your bridge; he has not set forth any plans by which he can obtain them. The English tube teeth, I think, have features of strength which you can not get with teeth with pins that are burned into the teeth, the latter being liable to fracture, probably from the effects of the fire, which is very annoying to all who have to do with bridge-work. But these English tube teeth are, I think, much stronger and the case is much easier repaired, and they give much less annoyance to the operator when repairs are necessary. I never have constructed any work in this manner, and therefore know very little about it, and have not much to say on the subject.

Dr. Wright, of Cincinnati: As there is another subject of a kindred nature to this, upon which Dr. Evans is to address us, I move that we hear Dr. Evans at this time, and then the discussion can be combined on the two papers—always provided that Dr. Evans is willing.

Motion seconded.

President Taft: Dr. Evans prefers that his paper be read to-morrow morning.

Dr. Wright: Then, with the permission of my second, I withdraw my motion.

Dr. Taft: If the subject is different, as I understand from Dr. Evans that it is, it will perhaps come in just as well to-morrow morning. We will continue the discussion on the present topic, and then proceed with the next paper. Are there any further remarks?

Dr. Harlan: I would like to ask Dr. Callahan, the author of the paper, one question. He says that in case of fracture of one of these teeth it is very easy to repair it. Now if he uses silver, the melting point of which is 268°, I don't think it is very easy to put one of those English tube teeth on the bridge in the mouth. You would have to melt the silver, or use oxy-sulphate of zinc, or some analogous substance; isn't that so?

Dr. Callahan: Yes, sir. In case you have to renew the crown, you would have to set it on with cement. I would not attempt to put on silver, although I have seen some mouths you could do it in, and it would not hurt them either.

Dr. Harlan: I only asked that question for fear somebody would get the idea that it would be easy to repair one of those bridges, and use silver, because they would have to apply more heat than could be borne by almost any patient.

The thing that strikes me as something that would be a great improvement in the making of bridges of this kind, is the manufacture of a half-round rim of gold in which to set the end of the ground tube tooth, so as to produce greater solidity, and which could be chamfered off to a minimum in appearance at the buccal aspect; that would add very much to the strength. Dr. J. H. Spaulding, of Paris, at the last meeting of the American Dental Association of Europe, read a paper on the use of English tube teeth in bridge-work, which was published in their transactions a short time ago, and in the *Dental Digest* and other publications, that has a pretty good set of directions for making this kind of bridges, which, added to Dr. Callahan's paper, will make a very complete treatise on that subject up to the present time. The remarks of Dr. Custer about a rest of pure gold or platinum under a bridge on the gum has the same objection that almost all appliances of that kind have, that is to say, they form a nest for micro-organisms; it is not food or saliva that causes the irritation, but it is the proliferation of micro-organisms, that produces redness, which is similar in kind to the redness that we find underneath rubber plates, wrongly attributed to the coloring matter of the plate.

Dr. Gillette, of Cincinnati: Dr. Ames gave his preference to diatoric over English tube teeth. The diatoric has a transverse opening, and I would ask him what disposition he makes of that? Does he leave it open, or fill it, or use it in the construction of the crown, making it stronger? And if he fills it, with what material; otherwise it would be uncleanly, it seems to me.

Dr. Ames: The transverse openings connect with the central chamber there, and in cementing, as I ordinarily do, that fills the lateral openings. I find that proper cementing saves plugging with gold. I had taken the precaution, in some cases, of plugging those little lateral openings with gold foil; but in those cases in which I did not, I find that the cement remained flush for several years, perhaps.

Dr. Taft: Are there any further remarks?

Dr. Ames: If I will be allowed I will say a little more. I omitted to say in regard to what I think we are coming to in this country in this class of work. I think the time is not far remote when we will have no use for English tube teeth, or diatoric teeth, used in this way. We will make solid bridges of porcelain right through, using, of course, some platinum to make our connection, attachment to the roots, and to give strength of span; but we will have very much more platinum or very much more porcelain embodied in the bridges of the next few years than we have in this.

President Taft: I would ask if Dr. Ames has any experience, or is it only imaginary?

Dr. Ames: I may say that I am adopting it to a greater extent right along, making molar crowns of porcelain, bicuspid crowns of porcelain almost entire, fitting a platinum band cap and dowel to that of the root; then taking what is ordinarily called a saddle-back tooth, which has pins. A saddle-back tooth is similar to a diatoric tooth, with the exception of having pretty much this shape (illustrating on blackboard). The saddle-back tooth, instead of having a chamfer, the shape of the diatoric, is more of a saddle-back, the pins coming out at about that angle (illustrating). Now, a bicuspid or molar can be adjusted in its proper position upon a platinum foundation, the band cap and dowel having been fitted to the root, and the pin bent down into contact with the platinum cap and soldered, then these places filled in with porcelain and baked either in—the only facilities I have of doing it at present being a Downey furnace, using Downey materials—this pin being usually allowed to approach as much as can be here; then bending the two pins around this, bending this pin down against the cap, as the case may be, filling it with porcelain and fusing it. I have confidence in that being a thoroughly substantial and the most artistic of anything possible to make. These saddle-back teeth, more especially for the molars, are to be obtained of better shape than either tube teeth or diatoric; when it comes to the molars you get the buccal surface and the masticating surface of an artistic shape, and all you need to do is to fill in this posterior surface, the getting of contour there being a very simple matter, and I must say I am very much in favor of it.

Dr. Wright: I want to call the attention of the Association to a remark of Dr. Harlan, and ask for some explanation in regard to it. Criticising Dr. Custer's remarks, he said there was no particular objection in those plates resting on the gum, to the saliva or food getting in there, but it was the micro-organisms. I should like to know what hurt a poor little micro-organism would do under a plate if there was no saliva or any meat and bread, or such things, there?

Dr. Harlan: Dr. Wright misunderstood me. The object of my remark was to cover the point that peripheral irritation of mucous membrane is the result of micro-organisms' presence. Of course they do find food for their sustenance in the saliva and other substances in the mouth; but the red spot that is usually there is caused by the excretion of the micro-organism, just as the red spots found under rubber and other plates are caused by the excretions of some organism, instead of being occasioned by the coloring matter or the presence of metal or rubber.

Dr. Wright: Then it is some specially active micro-organism that Dr. Harlan has a spite against.

Dr. Harlan: That is right.

Dr. Fletcher: I can report some failures. I have a liking to report failures. I have tried making porcelain bridges in the Downey furnace, with a platinum foundation. Last week I had a patient come in for whom I put on two bicuspids, using the canine for one abutment and the first molar for another. I had previously put on some bridge-work on the plan of using a crown covered with gold, fastening to the base, etc.—securely done. That broke down. I bethought myself of this plan, which, I adopted. Sent the patient away, telling him I thought now that was something he could not break; I don't think you can chew that to pieces. I did not see him until last week he came in with that bridge broken all to pieces. The platinum bridge itself had been made very strong, having been soldered with pure gold, and made very heavy and using all the porcelain that there was room to use, and the best material, building it out until I thought it was strong enough so that no teeth could break it. But that has been my experience. I have now another one that came back fractured. I saw it more than a month ago, it is made after the same plan, and that I think is going to do the same way. What the mistake is I do not know. But I thought it was the acme of strength so far as bridge-work goes. It has proved to be as much of a failure as I ever made, and I make a good many. If Dr. Ames or any other gentleman can enlighten us on that class of work I should be glad to hear it. It has been my plan for years to use very little gold on the teeth. A suggestion was made to me by Dr. Sherman. He takes a rubber tooth, gets rid of the head pins by pulling by punching with the pliers, and covers the teeth completely in the back with gold 22 carats, trimming it around the edge until it perfectly fits the back of it, and forms a shoe at the top, which Dr. Ames spoke of a few minutes ago. That is the first step, after you have selected the proper size. My plan is to run in 22 carat solder; then I fit the bridge, filling in with any lower carat solder you may see fit to use, also using platinum and iridium bars to strengthen it. In this way it don't show any gold at all, excepting at the gum margin. It forms a perfect shoe for the porcelain, the work being over the gold, so that it is perfectly protected in that way. If it breaks you cannot take it off and put it on new, as Dr. Callahan speaks of with the English tube crowns. At the same time I have very lately seen a case of bridge-work broken in a way that no one would imagine. The English tube crown does very well theoretically; if it pans out practically you could put on a crown accurately, but my experience is that these things break where you least expect it. I believe that the porcelain plan spoken of by Dr. Ames, if it be practical to put the body together in such a way that the process is available in an ordinary office, and you can get it sufficiently strong, it would be the acme of cleanliness; but the only thing it lacks is the strength, and that is one point I see against Dr. Ames porcelain-work.

Dr Ames: I don't think I claimed special strength, but artistic results, mostly.

President Taft: If there is no further discussion desired on this topic we will proceed with the regular order. Dr. Betty's paper would come next. Shall we take up that paper now? Has Dr. Callahan anything further to say?

Dr. Callahan: There is one point not touched upon, and that is the grinding of the teeth. If the English teeth are not exactly the shape you want you can grind them to any shape, then polish them, and have a perfect enamel. In regard to this sort of a bridge Dr. Custer spoke of, I don't think that was understood thoroughly. If you take a bridge in this shape and if the pressure is brought from below, the teeth being in position here, and folding this up somewhat, and if the pressure was below you can break it very easily but with the pressure from above down to the center here you could not break it at all, so far as the breaking of the metal is concerned. But if you break the porcelain off, as I say, it can be easily replaced, and there is no danger of breaking the pin, on account of the "V" shape. As to porcelain work being cleanly those of you who have taken old and continuous gum-plates and put them in the furnace and seen them smoke and smell, know they are not very cleanly.

On motion, further discussion on this subject was closed.

President Taft then announced that Dr. E. G. Betty, of Cincinnati, would read his paper on the "History of the Society."

